# Vein of Galen arteriovenous malformation with PAPVR and use of serial B-type natriuretic peptide levels in the management: a case report and review of the literature

**DOI:** 10.1186/1757-1626-3-43

**Published:** 2010-02-02

**Authors:** Isabell B Purdy, Nancy Halnon, Namrata Singh, Vladana Milisavljevic

**Affiliations:** 1 Department of Pediatrics, Division of Neonatology & Developmental Biology, David Geffen School of Medicine at University of California at Los Angeles, 10833 Le Conte Avenue, Room B2-375 MDCC, Los Angeles, CA 90095, USA; 2 Department of Pediatrics, Division of Pediatric Cardiology, David Geffen School of Medicine at University of California at Los Angeles, Los Angeles, CA 90095, USA

## Abstract

**Background:**

Arteriovenous malformation of the vein of Galen with partial anomalous pulmonary venous return can lead to a critically challenging condition associated with a high morbidity and mortality.

**Case report:**

We report a case of a full term infant born with a vein of Gallen arteriovenous malformation complicated by partial anomalous pulmonary venous return and congestive heart failure where B-type natriuretic peptide was used as a vital tool in clinical assessment and treatment management.

**Conclusions:**

Rapid diagnosis and treatment in infants with complex conditions such as this are imperative to expedite appropriate treatments, preventing long term negative outcome.

## Introduction

Vein of Galen arteriovenous malformations (VGAM) account for less than 1% of the arteriovenous malformations which are estimated to be seen in only 2.5 out of 100,000 live births [1]. VGAM consists of a saccular dilatation of the vein of Galen pooling blood shunted directly from abnormally enlarged cerebral arteries and often causing congestive heart failure (CHF), hypoxic respiratory failure, and/or persistent pulmonary hypertension of the newborn (PPHN).

Neonates born with VGAM who have complex cardiac disorders as well can present with pulmonary hypertension and/or CHF with additional management challenges. Even when echocardiography is easily available, a non-invasive screening test such as B-type natriuretic peptide (BNP) assay can be helpful in assessing and managing CHF in patients presenting with complex cardiopulmonary conditions.

BNP is secreted by myocytes primarily from the cardiac ventricles and directly correlates with left ventricular end diastolic pressure and wall stress. Monitoring of BNP in infants has proven to be a helpful non-invasive diagnostic and management screening tool [2,3]. While BNP assays have commonly been used to assess CHF in adult patients, it has only recently been used in neonates to differentiate cardiopulmonary diseases and to monitor the clinical course of such diseases with serial testing [3]. Infants with cardiovascular problems have significantly higher BNP levels (>550 pg/mL, range 578-1,435) compared to infants with non-cardiac problems that present with respiratory difficulties (mean 240 pg/ml, range 118-388) [4]. Healthy newborns tend to achieve a steady BNP level by 60 hours of life, with plasma concentrations highest on day of life (DOL) 0 (range approximately 56.7 +/- 49.6 pg/ml) and continuously decreasing, reaching adult levels at 3 months of age [5]. Past the neonatal period BNP levels should be less than 100 and before four months of age average around 21 pg/ml [6].

Cases of VGAM causing high output heart failure complicated by PPHN are rare and complex. A current literature review produced a few case reports on the pathological basis of PPHN leading to fatalities [7,8], rapid diagnostic imaging with ultrasound [9,10], and minimally invasive surgical techniques [11]. Tan et al. reported serial BNP levels following endovascular embolization for a neonate with uncomplicated VGAM and heart failure [12]. However, this report presents the first case study following serial BNP assays for management of care in a neonate with VGAM with high output cardiac failure complicated by partial anomalous pulmonary venous return (PAPVR) with sinus venosus defect and pulmonary hypertension.

## Case presentation

This full-term female infant was born via spontaneous vaginal delivery to a 32-year-old gravida 4, para 1 Caucasian mother at 40 and 5/7 weeks gestational age. Mother's medical history was unremarkable and pregnancy, labor, and delivery were uncomplicated. Birth weight was 3330 grams. Apgar scores were 8 at 1 and 5 minutes of age, respectively. After delivery, oxygen saturation was 93% and a 2/6 systolic murmur was noted. During a workup for infection and respiratory distress, oxygen saturation dropped to the 80s. Chest radiography showed cardiomegaly. Cranial ultrasound demonstrated a large vein of Galen arteriovenous malformation with MRI with contrast confirming the diagnosis. Dopamine was initiated for treatment of hypotension. Secondary to high flow AVM, this patient was monitored closely for increased risk for heart failure with serial BNP levels starting on DOL 4 when it was found to be 2450 pg/mL. Echocardiogram on DOL 6 showed high-output heart failure and the combination of a large sinus venosus ASD, with partial anomalous pulmonary venous return (PAPVR; right upper pulmonary vein to superior vena cava) and patent ductus arteriosus with pulmonary hypertension resulting in right to left shunting. On DOL 9, a cerebral angiogram and Vein of Galen aneurysm embolization were performed, reducing the flow to 50%. BNP level obtained on DOL 10 was over 5000 pg/mL. Post-procedure, patient was placed back on high frequency oscillator, inhaled nitric oxide (iNO) was started at 20 parts per million, and continued for 6 days. A second embolization treatment that significantly reduced the flow was performed on DOL 16. By DOL 21, BNP levels dropped to 426 pg/mL with pulmonary hypertension still evident on echocardiogram. At 3 months of age, cardiac catheterization showed significant blood return to the superior vena cava, likely a result of the VGAM, and pulmonary hypertension. Sildenafil was started and BNP levels dropped below 100 pg/ml, as the patient's condition improved. The patient was discharged home on oxygen, diuretics and albuterol. She was readmitted 1 week later for shortness of breath and tachypnea. BNP level was 218 pg/mL. She went into CHF quickly and required a third coil embolization at 5.5 months of age after BNP levels reached 2020 pg/mL.

**Figure 1:**
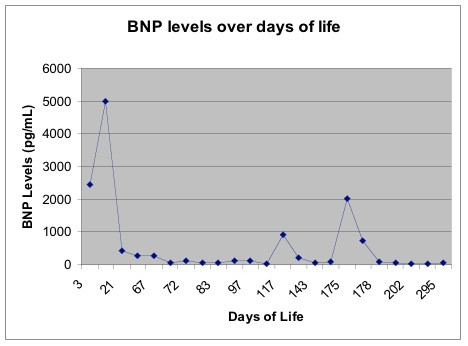
**Serial changes in BNP levels throughout patient's NICU and early pediatric hospital course**.

By six months of age, the patient required placement of a ventriculo-peritoneal shunt for hydrocephalus. She had one other brief hospitalization for an upper respiratory infection but remained stable and has not been hospitalized since. In no apparent respiratory or cardiovascular distress on DOL 295, her BNP level was 49 pg/mL. Figure 1 displays serial changes in BNP levels throughout her NICU and early pediatric hospital course.

Developmental evaluation at 46 weeks of age identified global functional developmental delays. However, at 22 months of age, her functional development was within normal. At the time of her last brain scan, no obvious shunting through the VGAM was detected.

## Discussion

During the neonatal period, symptomatic infants with VGAM present with severe cardiorespiratory alterations at or shortly after birth, with the majority of cases (94%) having high-output cardiac failure [13,14]. Severe PPHN may be a complicating factor. Since newborns classically develop CHF as a result of a tremendous left to right shunting through the low resistance vascular bed of the VGAM, symptomatic infants often appear cyanotic due to right ventricular volume and pressure overload [10].

Echocardiography holds an essential role in estimating ventricular function and shunting across the PDA and atrial septum, right ventricles and pulmonary artery pressures, and identifying associated cardiac conditions in infants with VGAM [13]. In addition, echocardiography may reveal reversal of aortic flow during diastole, also known as a steal phenomenon that decreases peripheral perfusion [10]. Cardiac failure and pulmonary hypertension are the most dramatic and challenging concerns for medical stabilization of the symptomatic neonate. NO, the most effective therapy for treating pulmonary hypertension, has limited success in infants with VGAMs and PPHN [10]. While optimal strategies are yet to be clearly defined, diuretic and inotrope therapy may be successful in helping deferral of endovascular treatment for these patients. However, management of those diagnosed with coexisting pulmonary hypertension is demanding and warrants serial BNP tests to monitor the clinical course.

The endovascular treatment of a VGAM often requires several successive procedures. Staged embolization sessions initially aimed at controlling cardiac failure help avoiding the occurrence of parenchymal bleedings or massive venous thrombosis potentially endangering the normal venous drainage. Monitoring beyond the neonatal period includes checking for increased head circumference, hydrocephalus, seizures, and developmental delays. The etiology of often associated psychomotor disabilities derives from the cerebral steal phenomenon [10].

Advances in endovascular techniques and perinatal management have led to more favorable long term outcomes [10,15]. A 30 month follow-up study reported that 55% of their patients were functionally normal [15]. While the past mortality rate for this population was close to 100% [16], more recent studies report 9-15% mortality and little to no neuromorbidity in 61-66% of survivors [17,18].

Rapid diagnosis and treatment in infants with complex conditions such as this are imperative to expedite appropriate treatments, preventing long term negative outcome. We speculate that including serial BNP levels in the arnament of assessment tools may aid the neonatal management and promote better outcomes in these complex patients.

## Abbreviations

VGAM: vein of Galen arteriovenous malformation; PAPVR: partial anomalous pulmonary venous return; BNP: B-type natriuretic peptide; NICU: neonatal intensive care unit; DOL: day of life; CHF: congestive heart failure; PVR: pulmonary vascular resistance; PPHN: persistent pulmonary hypertension of the newborn.

## Consent

Written informed consent was obtained from the patient for publication of this case report and accompanying images. A copy of the written consent is available for review by the Editor-in-Chief of this journal.

## Competing interests

The authors declare that they have no competing interests.

## Authors' contributions

IP and VM wrote the initial draft of the manuscript and helped revise it critically for important intellectual content. NH assisted with manuscript revision. NS have made substantial contributions to acquisition of data and have been involved in drafting the manuscript. All authors read and approved the final manuscript.
